# Artesunate attenuates the tumorigenesis of choroidal melanoma via inhibiting EFNA3 through Stat3/Akt signaling pathway

**DOI:** 10.1007/s00432-024-05711-8

**Published:** 2024-04-17

**Authors:** Ningning Yao, Qingyue Ma, Wendan Yi, Yuanzhang Zhu, Yichong Liu, Xiaodi Gao, Qian Zhang, Wenjuan Luo

**Affiliations:** Department of Ophthalmology of The Affiliated Hospital of Qingdao University, Qingdao, 266003 China

**Keywords:** Choroidal melanoma, EFNA3, Artesunate, Stat3, Akt

## Abstract

**Purpose:**

Choroidal melanoma (CM), a kind of malignant tumor, is the main type of Uveal melanoma and one half of CM patients develop metastases. As a member of Eph/ephrin pathway that plays vital role in tumors, EphrinA3 (EFNA3) has been proved to promote tumorigenesis in many tumors. But the effect of EFNA3 in CM has not been studied yet. Through inhibiting angiogenesis, inducing apoptosis and autophagy and so on, Artesunate (ART) plays a key anti-tumor role in many tumors, including CM. However, the exact mechanisms of anti-tumor in CM remain unclear.

**Methods:**

The UALCAN and TIMER v2.0 database analyzed the role of EFNA3 in CM patients. Quantitative real time polymerase chain reaction (qPCR) and Western blot were used to detect the expression of EFNA3 in CM. The growth ability of CM was tested by clonogenic assay and Cell counting kit-8 assay, and the migration ability using Transwell assay.

**Results:**

Our results found EFNA3 boosted CM cells’ growth and migration through activating Stat3/Akt signaling pathway, while ART inhibited the tumor promoting effect of CM via downregulating EFNA3. In xenograft tumor model, EFNA3 knockdown and ART significantly inhibited tumor growth.

**Conclusion:**

EFNA3 could be a valuable prognostic factor in CM.

**Graphical abstract:**

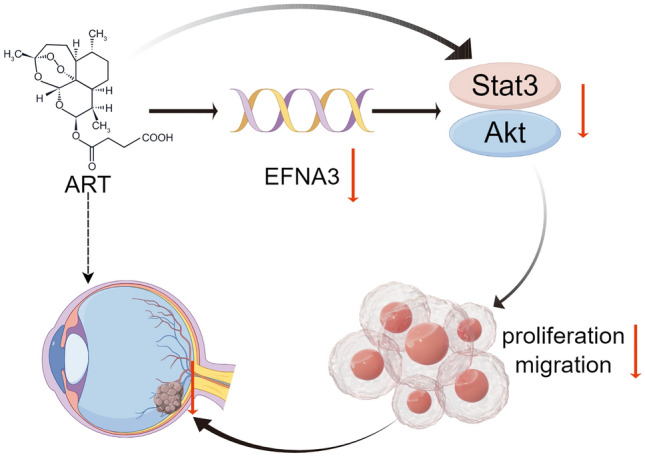

## Introduction

Choroidal melanoma (CM) is caused by malignancy melanoma cells composed of neuroectodermal tissues, origin from melanoma cells within the choroidal matrix, and is the predominant malignant tumors in adult eyes (Krantz et al. 2017). CM is easily transferred through the blood circulation with high degree of malignancy, and easily leads to blindness and death (Kaliki et al. 2015). More than one half of CM patients develop metastasis (Carvajal et al. 2017). Over the past 20 years, despite advances in combined treatments, for example surgery, radiotherapy, and laser therapy, the overall survival of CM patients remains poor (Smit et al. 2020; Rusňák et al. 2020; Wong et al. 2023). Therefore, it is necessary to continue explore the prognostic biomarkers and therapeutic targets for CM to provide more individualized therapies for a better prognosis.

The largest subfamily of receptor tyrosine kinases (RTKs), Eph/ephrin, plays a crucial role in multiple cellular processes of human body. The combination of Eph and Ephrins, its cognate ligands, could regulate cell function and biological process by bidirectional signals (Yiminniyaze et al. 2023; Kania and Klein 2016; Himanen and Nikolov 2003). Therefore, Eph/ephrin is considered as a promising therapeutic target in many diseases, including tumors. The different expression of EphrinA3 (EFNA3), a number of Eph/ephrin family, was related to the occurrence and development in variety of tumors (Zheng et al. 2021; Lin and Yang 2022; Yiminniyaze et al. 2023). What’s more, EFNA3 plays different roles in different malignancies, but its effect in CM not clear. Thus, our research aimed to elucidate the effect of EFNA3 in CM.

Artemisinin, extracted from the sweet wormwood plant, is a medication utilized in malaria treatment (El Ket et al. 2020). Following the success of Artemisinin in treating malaria, its other effects have been explored, and found that it can also inhibit the progression of cancers, inflammation and other diseases. In recent years, studies have focused on the anticancer effects of Artesunate (ART) which is a derivative of artemisinin. ART can play a cancer-suppressing role in many tumors by promoting apoptosis, autophagy, inhibiting cell proliferation and angiogenesis, etc., such as lung cancer, thyroid cancer and colorectal cancer (Wang et al. 2020a; Xu et al. 2022; Huang et al. 2022). ART shows promise as an anticancer drug by inhibiting many classical pathways. Recent studies have also indicated that ART can suppress the progression of CM (Geng et al. 2021; Farhan et al. 2021). However, the exact mechanisms remain unclear and more research is needed.

In this research, we discovered that EFNA3 promoted the tumorigenesis and activated Stat/Akt pathway in CM. We further discovered that ART suppressed the expression of EFNA3 and Stat3/Akt pathway. This finding may offer a novel potential therapeutic target of CM.

## Materials and methods

### Cell lines, cell culture, drug and data source

DMEM medium and RPMI-1640 medium with 10% FBS and 1% penicillin–streptomycin were used to incubate ARPE-19 and CM cells (C918, Ocm-1, Omm2.3 and Mel270), respectively. All cells were grown in humidified environment at 37 °C and 5% CO_2_. ARPE-19 and Ocm-1 were bought from Beijing BeNa Culture Collection, C918 from Wuhan Procell Life Science & Technology Co., Ltd, Omm2.3 and Mel270 from Guangzhou Cellcook Biotech Co., Ltd. ART were purchased from Sigma. In previous study (Geng et al. 2021), we examined the toxic effects of ART and the IC50 concentration in CM cells. The significance of EFNA3 in CM was analyzed using University of Alabama at Birmingham Cancer data analysis portal (UALCAN; https://ualcan.path.uab.edu/index.html) and tumor immune estimation resources (TIMER v2.0; https://cistrome.shinyapps.io/timer/).

### Quantitative real time polymerase chain reaction (qPCR)

According to instructions, RNA was extracted utilizing RNA-easy Isolation Reagent (Vazyme, Nanjing, China). Subsequently, RNA was reserve transcribed by HiScript III RT SuperMix for qPCR (+ gDNA wiper) (Vazyme, Nanjing, China) and amplified by qPCR. GAPDH was used to normalize the expression of target genes and the relative expression levels of gene were calculated according to the 2^−ΔΔCT^ method. h-EFNA3-F: CGCACAGCCCCATCAAGT, h-EFNA3-R: AGACGAACACCTTCATCCTCAGA.

### Western blot assay

Protein was isolated using RIPA with protease inhibitor and phosphatase inhibitors, separated using 10% SDA-PAGE, transferred to PVDF membranes, blocked with 5% nonfat milk at 37 °C for 1 h, and incubated with anti-GAPDH antibody (1:5000, Servicebio), anti-EFNA3 antibody (1:1000, HUABIO), anti-p-Stat3 antibody (1:2000, CST), anti-Stat3 antibody (1:2000, CST), anti-Akt antibody (1:1000, CST), anti-phosphor-Akt antibody (1:1000, CST) for overnight at 4 °C. Subsequently, PVDF was incubated with antibodies of secondary at room temperature for 1 h, detected with Sensitive ECL Luminescence Reagent (Meilunbio, Dalian, China).

### siRNA and plasmid transfection

siRNA of EFNA3 were purchased from GenePharma company (Shanghai, China). EFNA3 siRNAs sequences: siRNA #1,5′-CGUGAACGACUAUCUGGAUAU-3′; siRNA #2,5′-GCAGGUGAACGUGAACGACUA-3′. The overexpression plasmids pcDNA-EFNA3 were purchased from Genechem company (Shanghai, China). siRNA and plasmid were transfected into CM cell using Lipo8000^™^ transfection reagent (Beyotime Biotechnology, Shanghai, China) according to instructions.

### Assembly of EFNA3 lentivirus

First, suitable and transduction reagent were selected according to the requirements of manufacturer. Then cells were spread into 6-well plates and lentivirus was added into the plates when cells reached 20–30% confluence. Stable cells were screened using puromycin (1 μg/ml). EFNA3-downregulating lentivirus were bought from Genechem company (Shanghai, China).

### Cell counting kit-8 assay (CCK-8)

Two thousand cells were added in 96-well plates and cultured for overnight. When removing the original medium, 10 μ CCK-8 reagent (MCE, Weehawken, USA) and 90 μ RPMI-1640 was added. After incubation of two hours, microplate reader was utilized to assessed the absorbance at 450 nm wavelength. CCK-8 assay was performed for cultures at 0 h, 24 h, 48 h, 72 h, 96 h.

### Clonogenic assay

Each well was seeded 1000 cells in 6-well plates and cells were incubated with 10% FBS medium for 10–14 days. After 10–14 days, 4% paraformaldehyde was utilized to fix the colonies. Subsequently, crystal violet was employed to stained the colonies. Photoshop was used to count the number of colonies.

### Transwell migration assay

Cells in medium without FBS were added into upper chamber and medium containing 10% (C918) and 30% (Ocm-1, Omm2.3) FBS added into lower chamber as a chemoattractant. After being incubated for 24 h (C918) and 48 h (Ocm-1, Omm2.3), cells on the lower surface were fixed, stained, and counted. The assay was repeated thrice.

### Immunohistochemistry

Using 4% paraformaldehyde and paraffin to fixe and embed tumors, then cut into slices. After deparaffinization in dimethylbenzene, the slices were gradually dewatered in ethanol, treated with citrate buffer and immerged 3% hydrogen peroxide solution. Subsequently, slices were incubated using primary antibodies and secondary antibodies successively, and treated with diaminobenzidine, counterstained with hematoxylin.

### Nude mouse xenograft study

The animal experiments were approved by the ethics committee of the Affiliated Hospital of Qingdao University (permit number: AHQU-MAK20230526). The mouse experiment was in accordance with the institutional guidelines of Animal Care and Use Committee. Fifteen female BALB/c nude mice aged 4 weeks were bought from Jinan Pengyue. C918 cells (downregulating EFNA3 and empty vector control) were digested with pancreatic enzymes and resuspended in PBS counting 1/2 polymerized Matrigel to a final cell count of 2 × 10^7^/mL. A volume of 100 μ of the cell suspension was injected in the right armpit of the mouse. After one week, mice were administered solvent (the mouse of vehicle and shEFNA3), ART (i.p., 70 mg/kg, qd) for 14 days. The size of tumor was measured using digital caliper. At the conclusion of the experiment, the tumor was excised and subsequently weighed.

### Statistical analysis

All results were analyzed using *t*-teat (two groups) and one-way ANOVA (multiple groups) in GraphPad Prism 9.0 (GraphPad Software, USA). Data were presented as mean ± standard error of the mean (SEM). All experiments were performed at least in three times and *p* < 0.05 was regarded as significance (ns: no significance, *p < 0.05, **p < 0.01, ***p < 0.001, ****p < 0.0001).

## Results

### High EFNA3 expression correlated with bad prognosis of CM

**Fig. 1:**
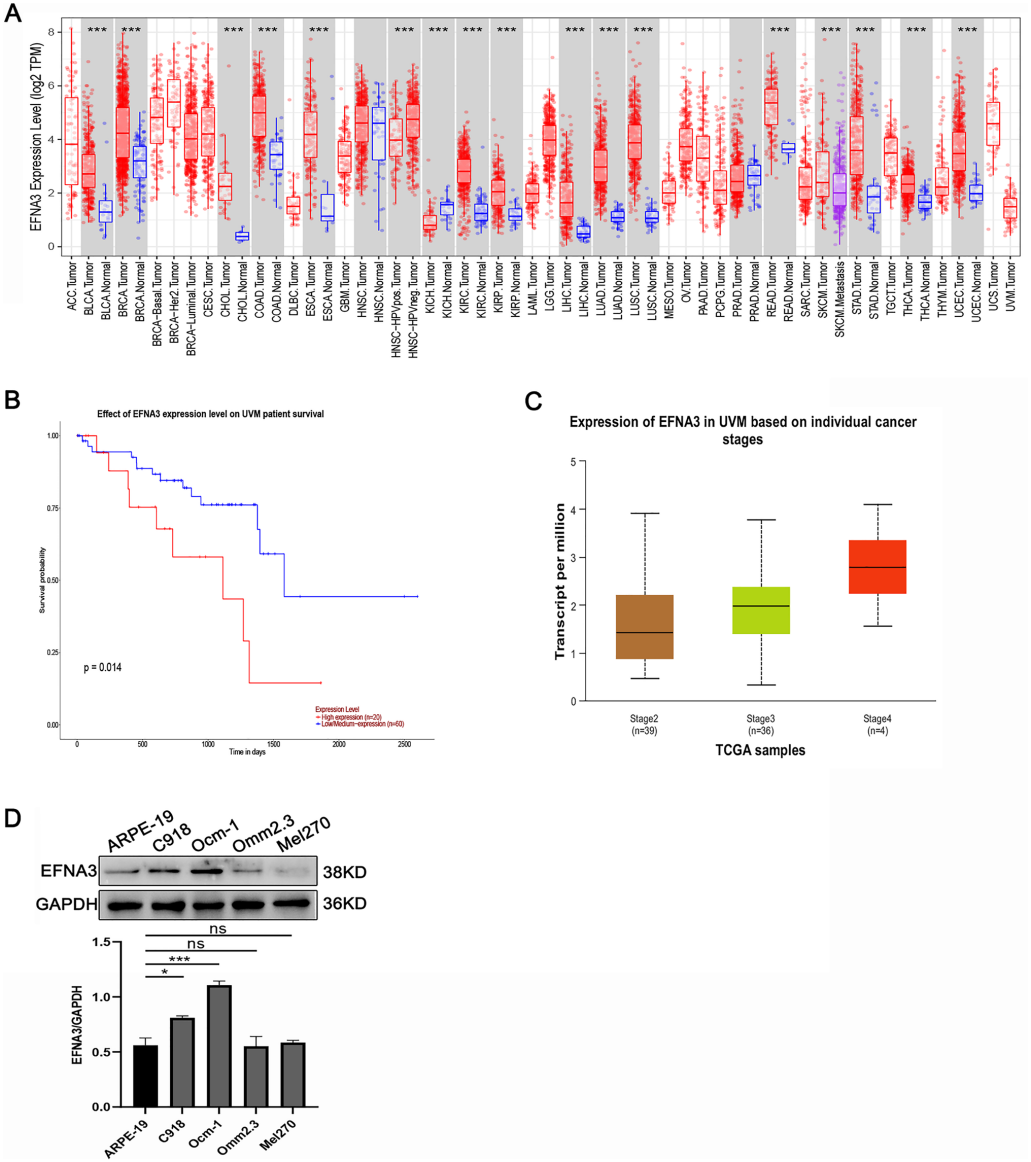
High level of EFNA3 correlated with poor prognosis of CM. **A** TIMER database was employed to detect the expression of EFNA3 in various tumors. **B** High level of EFNA3 correlated with poor prognosis of CM patients (*n* = 80). **C** Expression of EFNA3 in CM patients based on cancer stages. **D** The protein expression levels of EFNA3 in CM cell lines

TIMER database proved that EFNA3 was differentially expressed and significantly high expressed in many tumors, including CM (Fig. 1A). Moreover, UALCAN database found high level of EFNA3 was significantly associated with bad overall survival (Fig. 1B). We also founded the higher level of EFNA3 was, the higher tumor malignancy degree of CM patients was (Fig. 1C). To further validation, we used Western blot to detect EFNA3 protein levels and discovered the level of EFNA3 was higher than ARPE-19 (Fig. 1D). Above results suggested that EFNA3 may contribute to the development of CM.

### EFNA3 enhanced the proliferation of CM cell lines

**Fig. 2:**
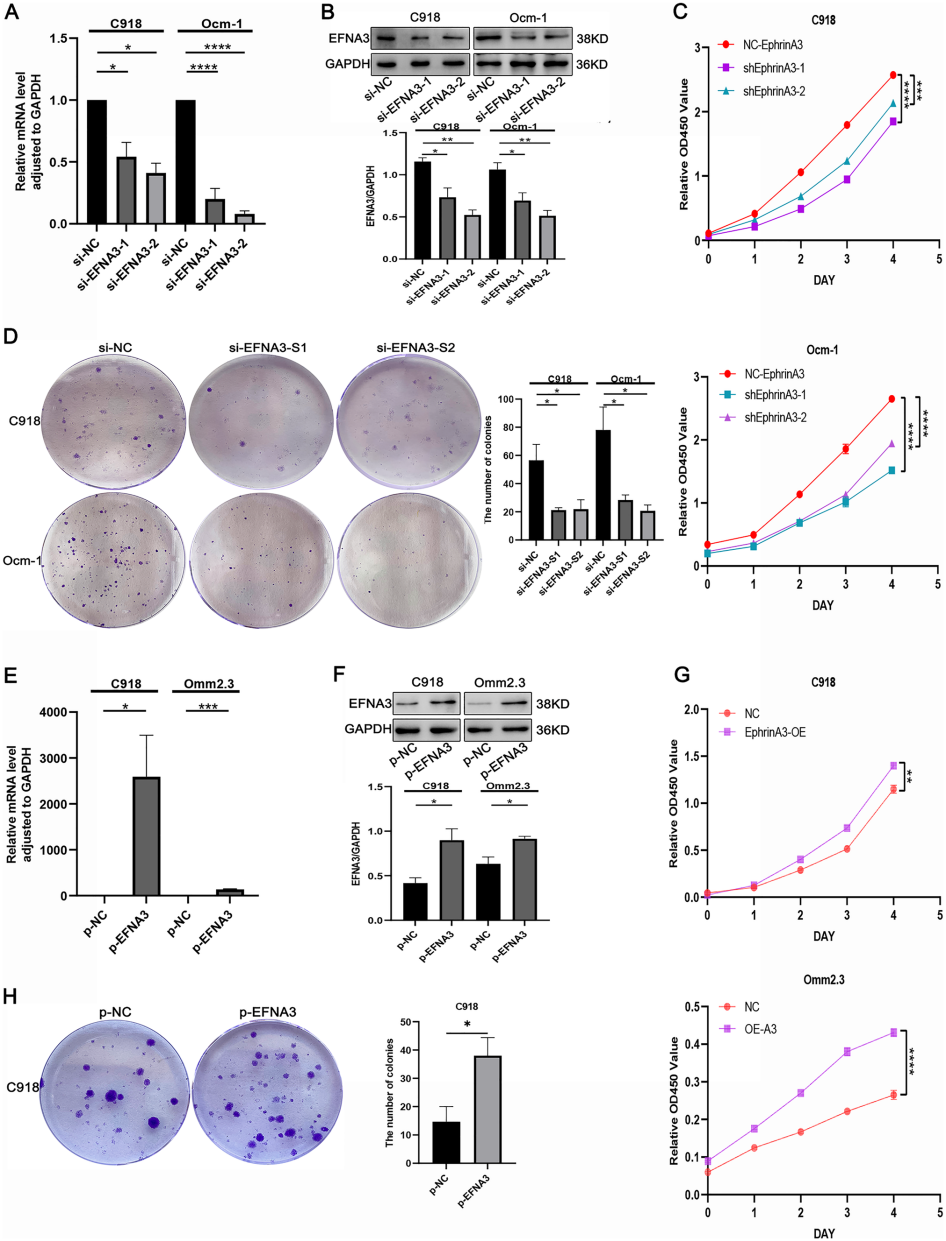
EFNA3 enhanced the growth of CM cells. **A**, **B** Knockdown of EFNA3 with siRNA in C918 and Ocm-1 (*n* = 3). **C**, **D** EFNA3 knockdown inhibited proliferation of CM cells according to CCK-8 and Clonogenic assay (*n* = 3). **E**, **F** Upregulation of EFNA3 in C918 and Omm2.3 cells (*n* = 3). **G**, **H** EFNA3 overexpression promoted growth of CM cells (*n* = 3)

We inhibited the expression of EFNA3 with small interfering RNA (siRNA) technology in C918 and Ocm-1 to investigate the role of EFNA3, and verified using qPCR and western blot (Fig. 2A, B). Knockdown of EFNA3 damaged the growth of CM cells (Fig. 2C, D). We also overexpressed EFNA3 in CM cell lines, C918, and Omm2.3, to further investigate the role of EFNA3. The efficiency of transfection was verified by qPCR and western blot (Fig. 2E, F). As shown in CCK-8 assay, the OD values were significantly higher in the cells of overexpression EFNA3 than vector group (Fig. 2G). Clonogenic assay showed that CM cells’ growth ability was significantly increased compared with vector group (Fig. 2H). Together, EFNA3 increased the growth of CM cells.

### EFNA3 promoted the migration ability of CM cells

**Fig. 3:**
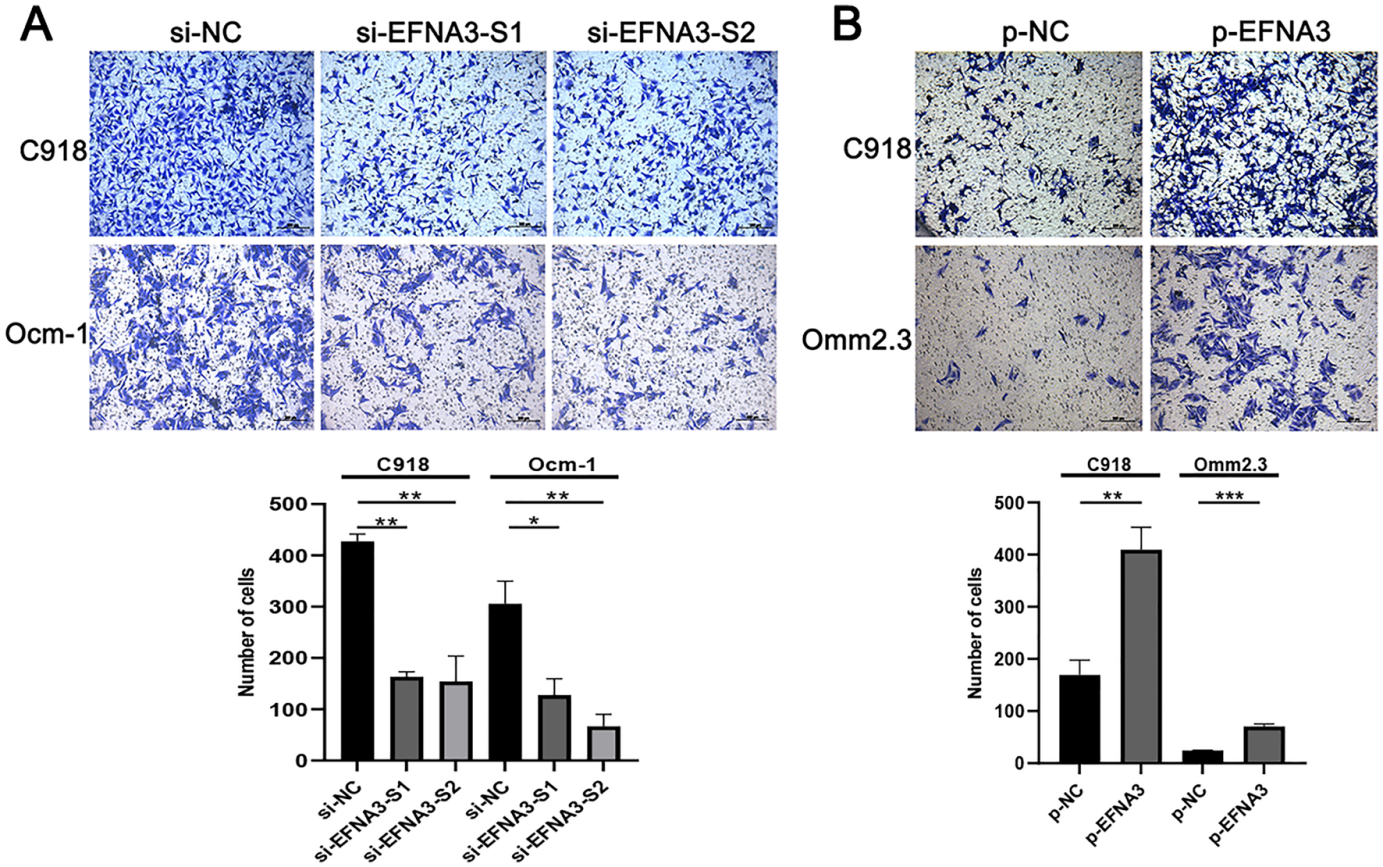
EFNA3 promoted CM cells’ migration ability. **A** EFNA3 knockdown inhibited migration of CM cells according to Transwell assay (*n* = 3). **B** EFNA3 overexpression promoted migration of CM cells (*n* = 3). Scale bar, 200 μm

The role of EFNA3 on the migration of CM cell lines was further investigated. The ability of migration was examined using Transwell assay. Downregulation of EFNA3 levels weakened the migration ability of CM (Fig. 3A) as shown in Transwell assay. What is more, the migration ability of CM cells was significantly promoted when overexpression EFNA3 (Fig. 3B). In conclusion, EFNA3 promoted CM cells’ migration ability.

### EFNA3 activating the Stat3/Akt signaling pathway in CM cells

**Fig. 4:**
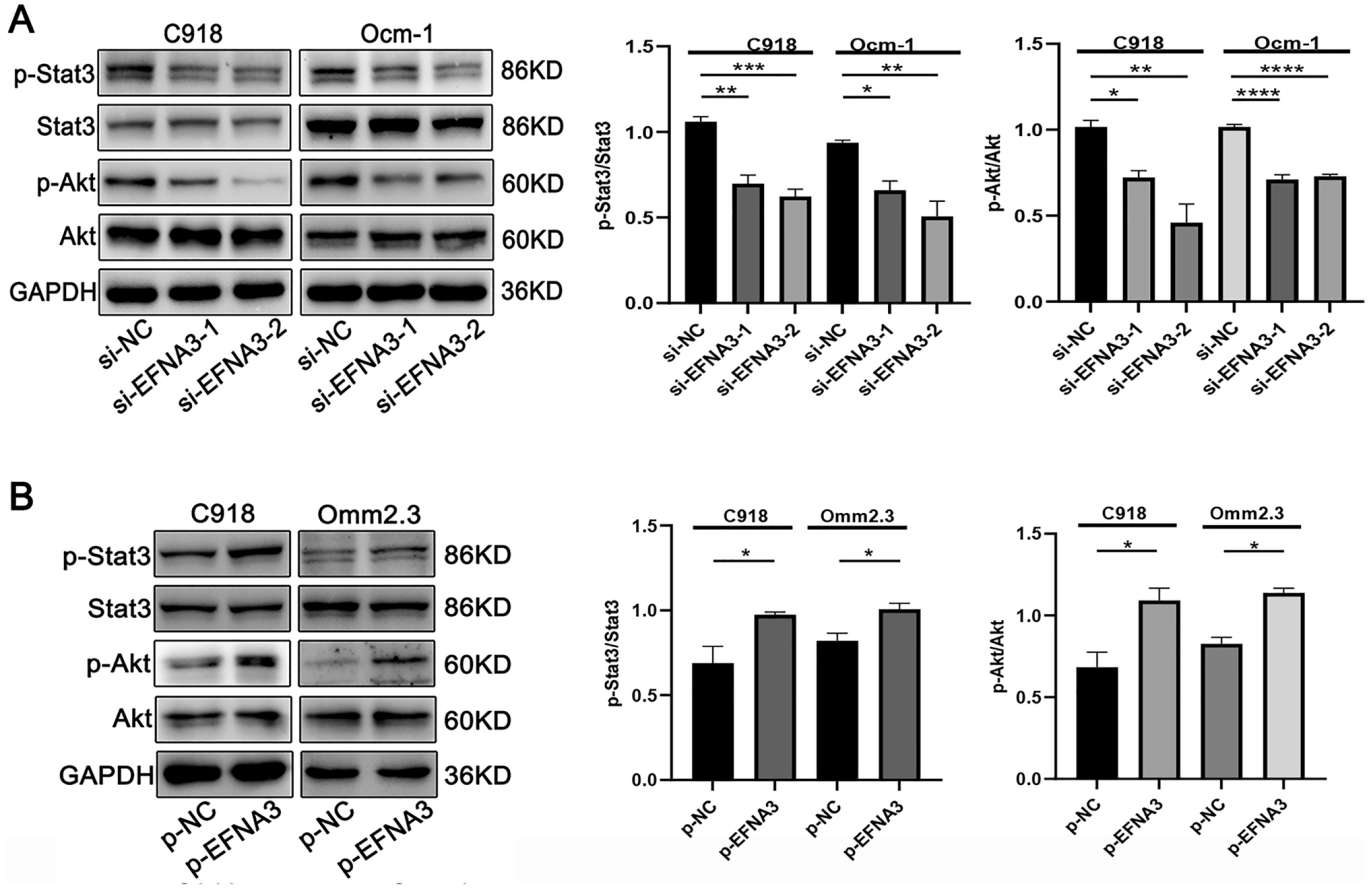
EFNA3 activating Stat3/Akt signaling pathway in CM cell lines. **A** EFNA3 knockdown inhibited p-Stat3 and p-Akt, while **B** its overexpression promoted p-Stat3 and p-Akt (*n* = 3)

Based on the critical role of EFNA3 in the development of CM, we next studied the molecular mechanism of EFNA3 promoting CM cell proliferation and migration. Transcription factor stat3 is involved in cellular inflammation, tissue repair and tumorigenesis (Hu et al. 2019; Tolomeo and Cascio 2021; Yu et al. 2009). Akt pathway also plays a critical role in the tumorigenesis of various tumors (Aoki and Fujishita 2017). Therefore, we detected the relationship between EFNA3 and Stat3/Akt pathway and found that EFNA3 knockdown inhibited the p-Stat3 and p-Akt, while EFNA3 overexpression enhanced the activation of Stat3/Akt pathway (Fig. 4A, B). Together, these data suggest that EFNA3 activating Stat3/Akt signaling pathway in CM cells.

### ART inhibited the proliferation and migration through inhibiting EFNA3 in CM cell lines

**Fig. 5:**
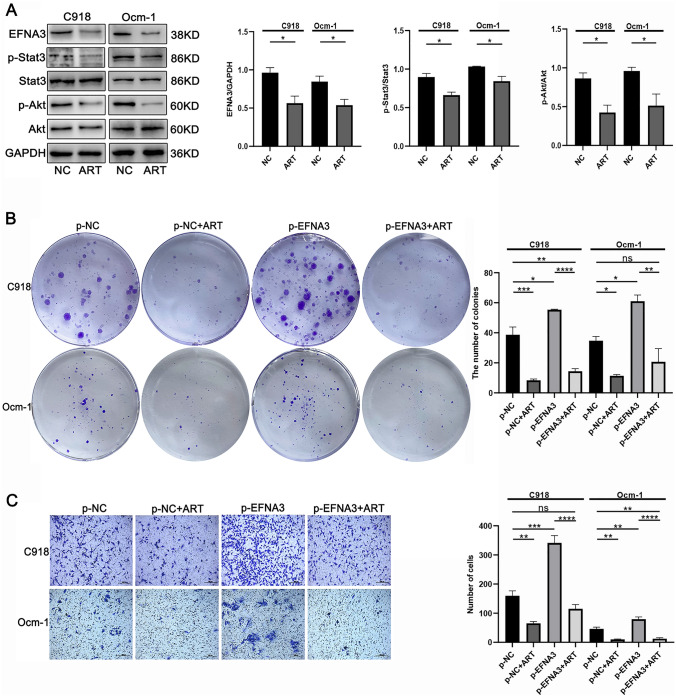
ART inhibited the proliferation and migration through inhibiting EFNA3 in CM cell lines. **A** ART (50 μm, 24 h) inhibited EFNA3, p-Stat3, and p-Akt (*n* = 3). **B**–**C** Clonogenic assay and Transwell assay results in ART (C918:30 μm, Ocm-1:10 μm, 24 h) treatment group with/without upregulation EFNA3 group (*n* = 3). Scale bar, 200 μm

ART plays a key role in anticancer effect in many cancers, including Choroidal Melanoma (Geng et al. 2021). For CM, ART is a promising therapeutic strategy. Above founding illustrated that EFNA3 promoted CM cells’ proliferation and migration ability, so we speculated ART inhibit the expression of EFNA3. To explore the role of ART on the expression of EFNA3, C918 and Ocm-1 with highly basic expression of EFNA3 were treated with ART. Western blot analysis demonstrated that ART significantly suppressed EFNA3, p-Stat3 and p-Akt in CM (Fig. 5A).

To further probe the effect between ART and EFNA3, we carried out rescue experiments on ART and EFNA3. The reduced proliferation capacity of CM cell in the ART group was restored after the overexpression EFNA3 (Fig. 5B). Transwell migration assay showed the same trend (Fig. 5C). Thus, these results displayed that ART suppressed CM cells proliferation and migration capacity through suppressing the expression of EFNA3.

### Both ART and downregulating EFNA3 inhibited tumor growth in vivo

**Fig. 6:**
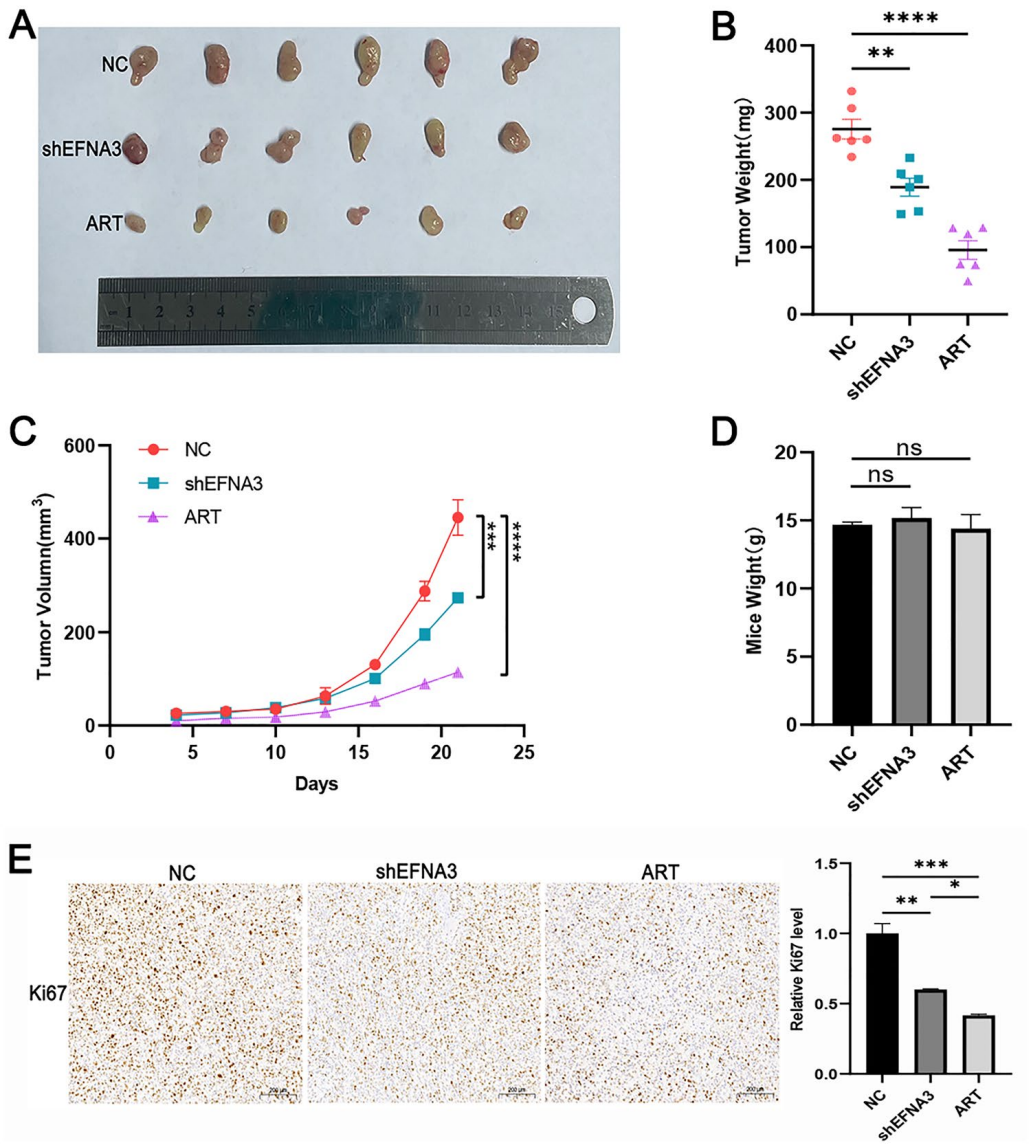
ART (i.p., 70 mg/kg, qd, 14 days) and downregulating EFNA3 both inhibited tumor growth in vivo. **A**–**D** The subcutaneous tumor images, growth curve, tumor weight and mice weight revealed ART application and knockdown EFNA3 both inhibited tumor growth in vivo. **E** Tumor cell proliferation statue was analyzed by IHC staining for Ki67. Scale bar, 200 μm

Finally, the role of EFNA3 and ART was examined in murine subcutaneous xenograft model with three groups: control, knockdown EFNA3 and ART group. We discovered that the size and weight of tumor in ART treatment group were smaller than control group and knockdown EFNA3 group (Fig. 6A–D). What is more, knockdown EFNA3 group were smaller than control group. Ki67 the tumor proliferation index was decreased in knockdown EFNA3 and ART treatment group (Fig. 6E). All data indicated ART inhibited CM growth in vivo through suppressing EFNA3.

## Discussion

CM patients face a highly unfavorable prognosis and usually suffer from a disease burden. The process of tumorigenesis is very complicated, and many pathways are involved (Wang et al. 2020b; Xia et al. 2023; Hu et al. 2019; Schmitt et al. 2022). There has been only a handful of advancements in the treatment of CM in the past 20 years. Therefore, gaining further insights into the underlying mechanism of CM tumorigenesis may reveal innovative targets for effectively managing CM tumors.

Eph/ephrin signaling plays a vital role in many physiological aspects of human body, including axon orientation of nervous system, angiogenesis, vascular remodeling and homeostasis of various tissues (Pasquale 2008; Cramer and Miko 2016; Nikas et al. 2022). According to the structure and sequence conservation, Eph receptor has been divided into two subtypes EphA (EphA1-EphA8, EphA10) and EphB (EphB1-EphB4, EphB6) (Surawska et al. 2004), and the ligands Ephrins also have two subtypes EphrinA (EphrinA1-EphrinA5) and EphrinB (EphrinB1-EphrinB3) (Kaczmarek et al. 2021). In Eph/ephrin signaling, EphA2, EphB4 and EphrinB2 have been extensively studied especially in tumor. But in recent years, there has been many researches about EFNA3, and its role is diverse in cancers. EFNA3 plays a pro-tumor role in lung cancer (Yiminniyaze et al. 2023), while an anticancer effect in oral squamous cell carcinoma (Wang et al. 2020b) and peripheral nerve sheath tumor (Wang et al. 2015). The role of EFNA3 in different tumors may be related to the tumor environment. However, there are no researches on the relationship between EFNA3 and CM. Our present data in UALCAN database showed that when EFNA3 expression is up-regulated, the prognosis of CM patients is poor, indicating the important significance of EFNA3 pathogenesis of CM. The growth and migration of CM cells were promoted by EFNA3 overexpression, and reversed by knockdown EFNA3. A previous study consistently demonstrated cancer cells exhibiting slow-cycling behavior tended to form larger colonies and displayed enhanced self-renewal capacity in vitro (Roesch et al. 2010). The low-density cell growth test utilized in the study shares several characteristics with the dissemination of tumor cells (DTCs) in secondary colonization sites, where cells remain quiescent before proliferating when exposed to favorable nutrient conditions (Crea et al. 2015; Guereño et al. 2020). This observation could also explain the augmented migration ability of CM cells with EFNA3 overexpression as in Transwell assays. Therefore, our findings demonstrate that EFNA3 has important effect in CM. The prognostic and therapeutic value of EFNA3 in UM patients and other tumor types worth in-depth research.

The different role of EFNA3 in various tumor may be related to the distinct background of intracellular signaling networks. In lung adenocarcinoma (LUAD), EFNA3 induces EMT by regulating ERK and p38MAPK pathway to affect the growth and metastasis of LUAD cells (Yiminniyaze et al. 2023). In hepatocellular carcinoma (HCC), EFNA3, promoted by HIF1-α under hypoxia conditions, affected the self-renew, proliferation and migration of HCC cells (Husain et al. 2022). We found that EFNA3 regulated cell ability and migration by regulating the levels of EFNA3, while the mechanism of how EFNA3 regulated tumorigenesis of CM need further research. The quiescence and activation of cancer cells involve a variety of intracellular signaling pathways, including the Stat3 and Akt signaling pathway. Stat3 and Akt signaling pathway play a key role in the progression of various cancers (Ma et al. 2020; Yu et al. 2014; Aoki and Fujishita 2017; Alzahrani 2019). Moreover, EFNA3 was reported can regulate the Stat3 and Akt pathway signaling (Xu et al. 2023; Wang et al. 2020b). In the mechanistic study, we discovered that EFNA3 knockdown inhibited Stat3/Akt pathway, while EFNA3 overexpression enhanced the activation of Stat3/Akt signaling pathway. Therefore, EFNA3 may promote the tumorigenesis of CM by regulating Stat3/Akt signaling.

ART, a Chinese herbal medicine, regulates many cellular processes and has been proved to be a potent anticancer drug in many tumors has been proved (Jia et al. 2023; Xu et al. 2022; Li et al. 2021). Studies found ART regulated Eph/ephrin signaling pathway in different cancers. In CM, ART significantly inhibited the expression of EphA2 (Geng et al. 2021). In breast cancer, ART increased EPHA8, EPHA10, EFNA2 and reduced EPHA3, EPHA7, and EFNA3 (Zadeh et al. 2022). However, it is not clear whether ART regulate the level of EFNA3 in CM. Our previous studies have proved that ART inhibits the tumorigenesis of CM, so we speculated ART plays a role in inhibiting CM prognosis by regulating EFNA3. In this research, we treated CM cells with ART and found that ART suppress EFNA3 and Stat3/Akt pathway. The rescue experiments showed that overexpression of EFNA3 partially improved the growth and migration ability which inhibited by ART in CM cells, suggesting that ART inhibit the tumorigenesis of CM by regulating EFNA3. Considering that overexpression EFNA3 in ART-treated CM cells reversed ART-induced inhibition of proliferation and migration, these results suggested that ART inhibited proliferation and migration by interacting with EFNA3 in CM.

In summary, EFNA3 is an important oncogene in CM. Knockdown of EFNA3 inhibited CM cell proliferation and migration in vitro and tumor growth in vivo. ART inhibited CM prognosis through regulating EFNA3 expression. Mechanistically, EFNA3 suppressed Stat3/Akt signaling pathway, and ART regulates tumorigenesis by inhibiting EFNA3 and it inhibited Stat3/Akt signaling pathway. Our findings showed new insight on the role of EFNA3 in tumorigenesis and provide new targets for the treatment of CM.

## Data Availability

The datasets generated during and/or analysed during the current study are available from the corresponding author on reasonable request.
